# Supplementary material for: Investigation of Salmonella Phage–Bacteria Infection Profiles: Network Structure Reveals a Gradient of Target-Range from Generalist to Specialist Phage Clones in Nested Subsets
Source: Viruses. 2021 Jun 28;13(7):1261. doi: 10.3390/v13071261 (PMC8310288; doi:10.3390/v13071261)
Supplement: Supplementary file 1 [file viruses-13-01261-s001.zip › viruses-1239528-si final.pdf]

Supplementary material includes Supplementary Figures 1-5, Supplementary Tables 1 and 2, and the following data files:

- “Supplementary Data File 1.csv”,
- “SalmonellaPBIN.Rmd”,
- “salmonellaPBIN.csv”, and
- “Supplementary Data File 2.

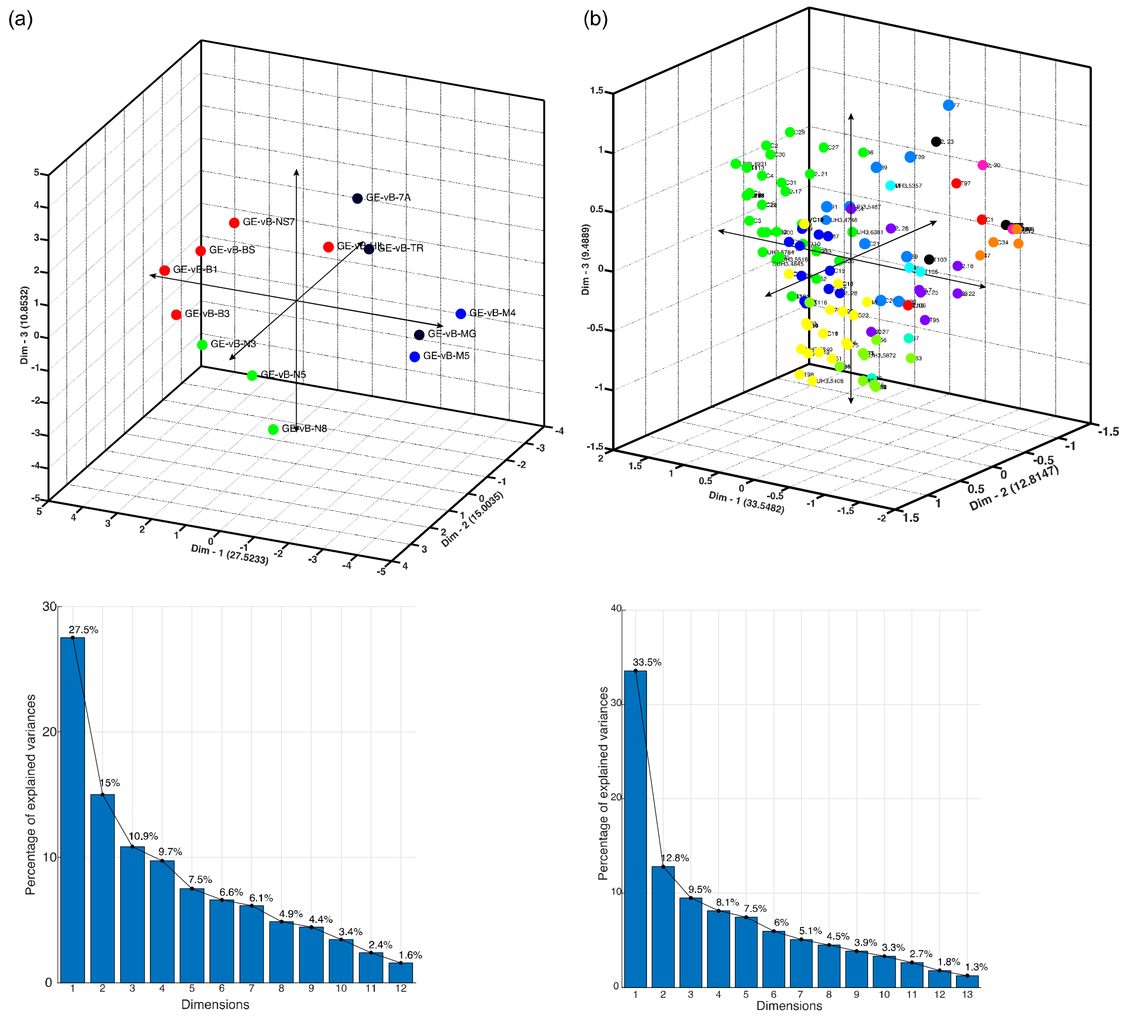

Supplementary Figure 1: Principal component analysis of the *Salmonella* phage-bacteria infection profiles.

Based on the infection profiles of the *Salmonella* phages (a) and the *Salmonella* bacteria (b), the Jaccard distances (dissimilarity) amongst the different species were calculated and annotated on principal components (PCA). The PCA decomposition is

presented in ranked bar plots which describe the percentage of the explained variance in different dimensions. Viral and microbial species are colour-coded based on the infection clusters that were assigned to using unsupervised hierarchical agglomerative clustering (see Figure 2); For phages: Red: cluster 1, Black: cluster 2, Green: cluster 3, and Blue: cluster 4.

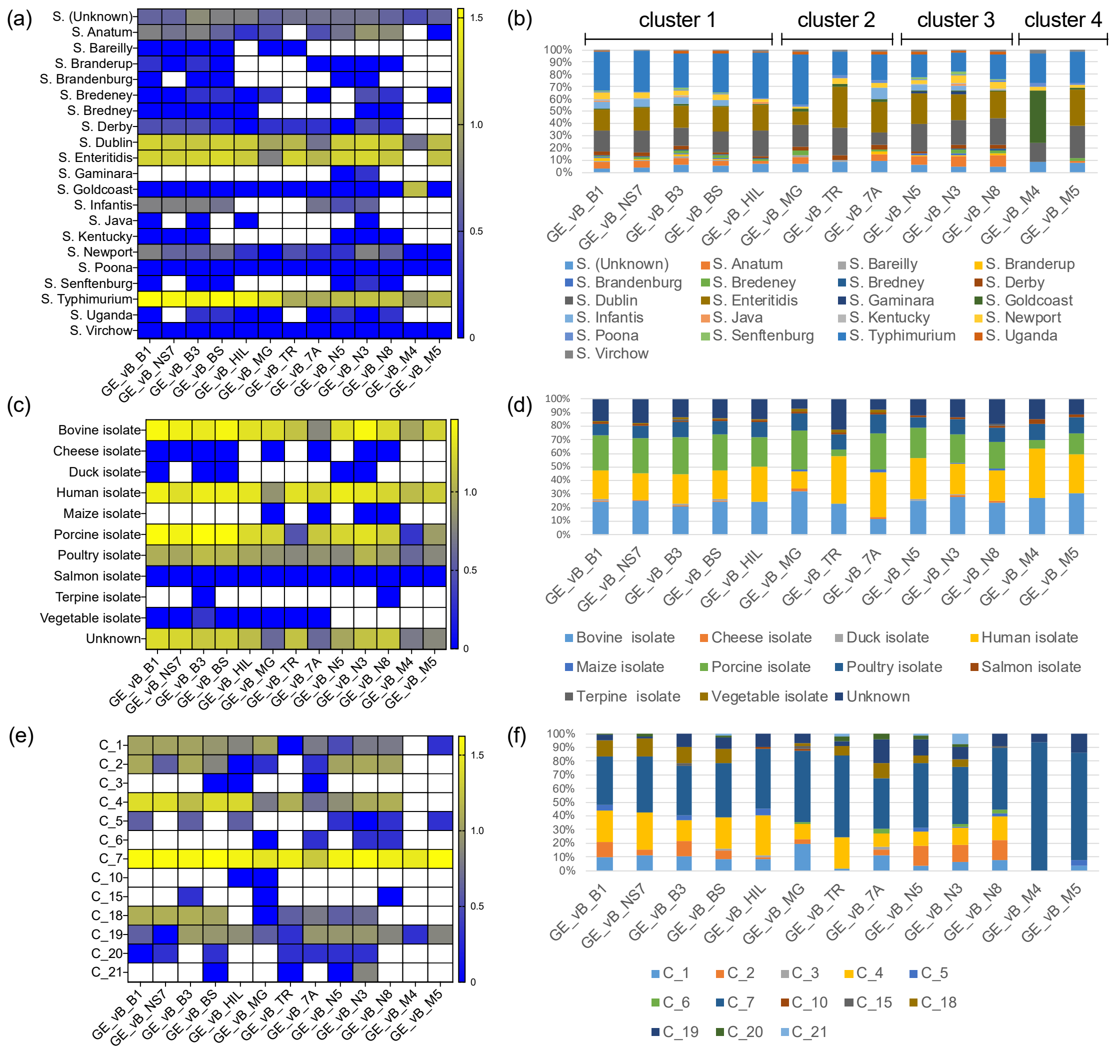

Supplementary Figure 2: *Salmonella* phage infection profiles.

The infection profiles for each one of the *Salmonella* phages included in this study is presented based on three factors: (a-b) The *Salmonella* bacteria species that were infected, (c-d) the type of environmental isolates of the bacteria, and (e-f) the infection cluster that the bacteria belonged to. For each factor the absolute number of infected *Salmonella* strains is presented in a heatmap format (a, c, & e), as well as stacked bar plots representing compositional infection profiles (b, d, & f). The phage compositional infection profiles are grouped according to the infection cluster that they were assigned to. Heatmap colour is analogous to the log_{10} transformed number of absolute counts of successful infections (High; yellow to Low; Blue).

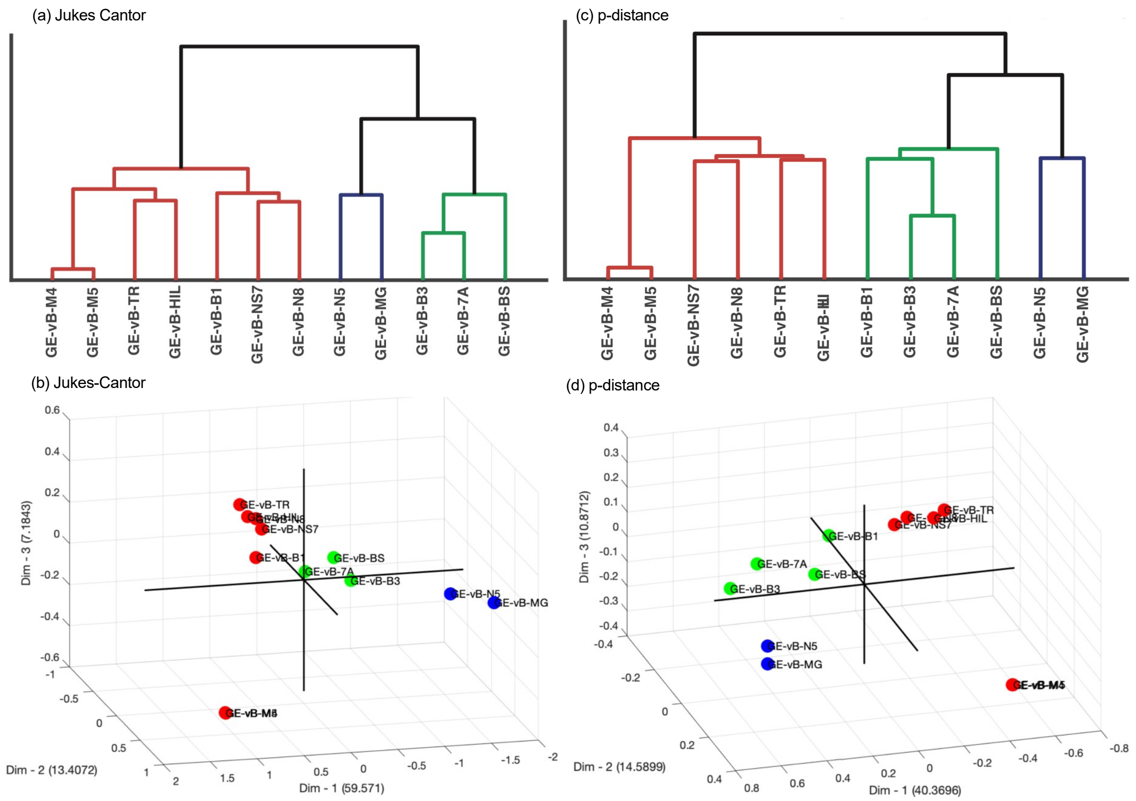

Supplementary Figure 3: *Salmonella* phage genetic distances

The complete genome sequences of twelve out of the thirteen Salmonella phages were used to measure their pairwise genetic distances (Jukes-Cantor and p-distance). For each distance measure, the phages were grouped using hierarchical clustering (complete linkage) and annotated in principal components: (a) and (b) for Jukes-Cantor, (c) and (D) for p-distance. Both distance measures produced similar results.

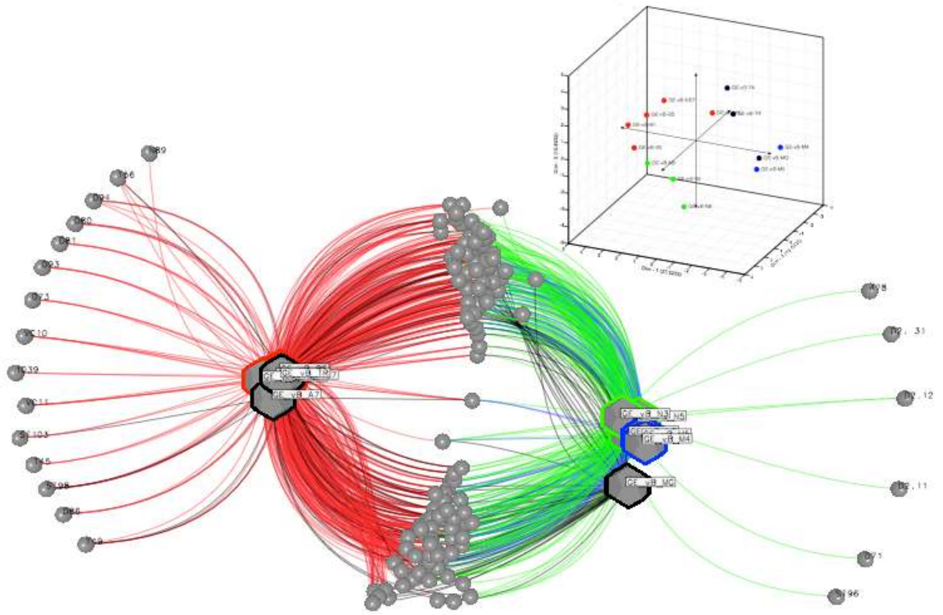

Supplementary Figure 4: *Salmonella* phage-bacteria infection network.

Three-dimensional network representation of the *Salmonella* phage-bacteria prey-predator ecological interactions. Polygonal nodes represent phages, and spheres bacteria. Edges connect phage species with their infected bacterial hosts. Phages are coloured based on the infection cluster that they were assigned to (see Figure 2). Red: cluster 1, Black: cluster 2, Green: cluster 3, and Blue: cluster 4. Similarly, the edges are also coloured based on the infection profile cluster of the phages. The network is

organised into one component. A GRIP algorithm was used to layout the network (Graph Drawing with Intelligent Placement). The observed “polarity” of the network is in line with the horizontal phage displacement of the *Salmonella* phages according to the dissimilarity of their infection profiles (PCA plot).

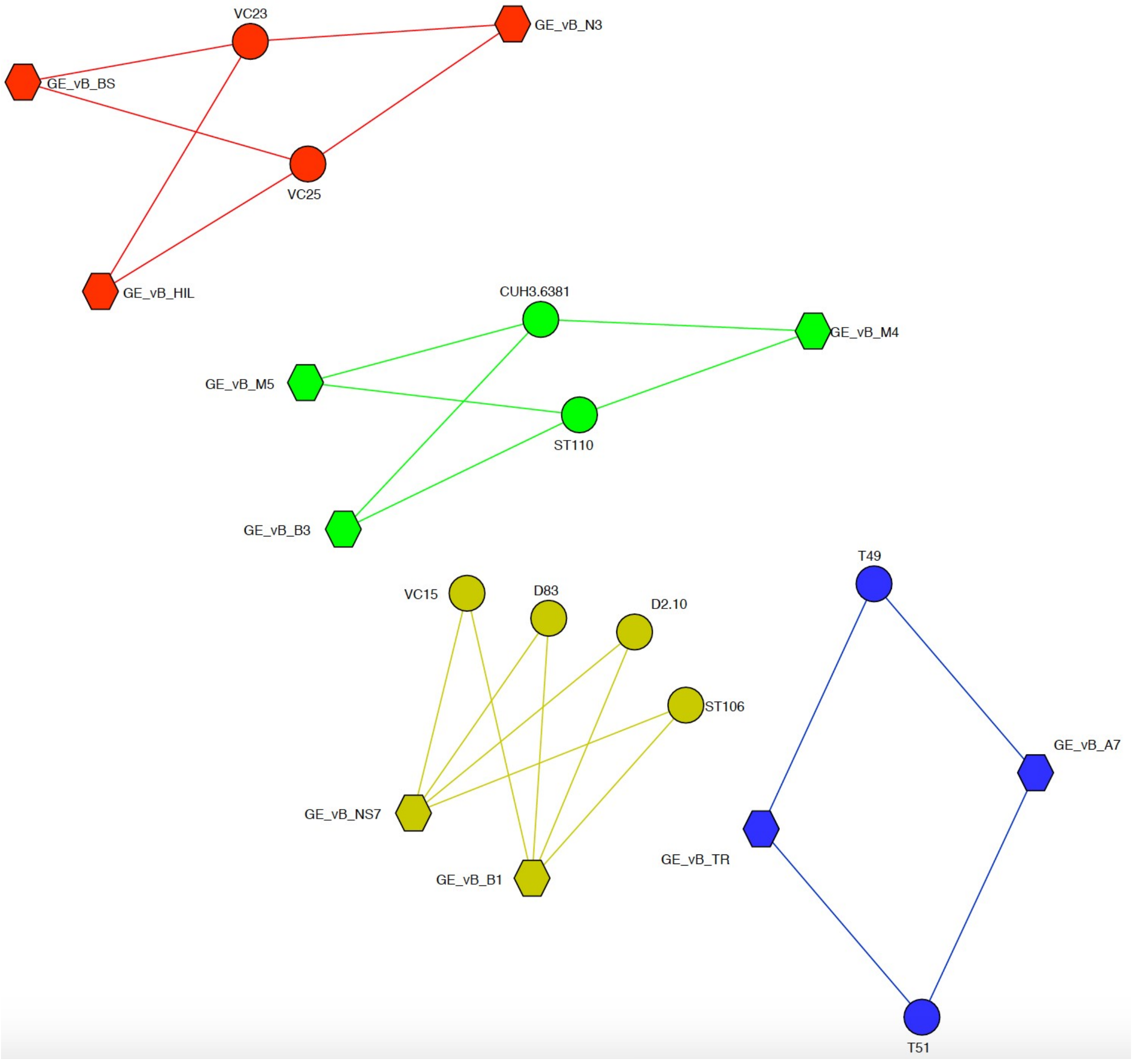

Supplementary Figure 5: Cliques within the *Salmonella* phage-bacteria infection network. Cliques represent sub-clusters within the network; Four different cliques were identified, which might reflect increased speciation amongst these phage-bacteria

species. Clique 1: Red nodes, Clique 2: Green nodes, Clique 3: Yellow nodes, Clique 4: Blue nodes

NN	Phage	Family	Sub-family	Genus	Sequencing	Accession
1	GEC_vB_B1	Myoviridae	Ounavirinae	Felixounavirus	NGS	MW006474.1
2	GEC_vB_B3	Myoviridae	Ounavirinae	Felixounavirus	NGS	
3	GEC_vB_NS7	Myoviridae	Ounavirinae	Felixounavirus	NGS	MW006482
4	GEC_vB_BS	Myoviridae	Ounavirinae	Felixounavirus	NGS	MW006475.1
5	GEC_vB_MG	Myoviridae	Vequintavirinae	Seunavirus	NGS	MW006477.1
6	GEC_vB_7A	Myoviridae	Markadamsvirinae	Felixounavirus	Nanopore	
7	GEC_vB_N3	Siphoviruses/Demerecviridae	Markadamsvirinae	Tequintavirus	NGS	MW006478
8	GEC_vB_N5	Siphoviruses/Demerecviridae	Markadamsvirinae	Tequintavirus	NGS	MW006479.1
9	GEC_vB_N8	Siphoviruses/Demerecviridae	Markadamsvirinae	Tequintavirus	NGS	
10	GEC_vB_M4	Siphoviridae	Guernseyvirinae	Jerseyvirus	Nanopore	
11	GEC_vB_M5	Siphoviridae	Guernseyvirinae	Jerseyvirus	Nanopore	
12	GEC_vB_HIL	Siphoviridae	Guernseyvirinae	Jerseyvirus	Nanopore	
13	GEC_vB_TR	Podoviridae	N/a	Lederbergvirus	Nanopore	

Supplementary Table 1: Description of the Salmonella phages used in this study.

This includes the NCBI taxonomy phage identification, the taxonomic lineage (Family, Sub-family, Genus), the sequencing technology used for molecular typing, and the NCBI accession numbers (GenBank) corresponding to the complete genome sequences, if available.

Hosts	140
Phages	13
Interactions	1028
Species	153
Size	1820
Connectance	0.56
Interactions across hosts	7.34
Interactions across phages	79.08

Supplementary Table 2: Basic characteristics of the *Salmonella* PBIN.

These include: number of host (H), number of phages (P), number of interactions (I), number of species ($S=H+P$), size ($M=HP$), connectance ($C=I/M$), mean number of interactions across host strains ($L_H= I/H$), and mean number of interactions across phage species ($L_P= I/P$).

Supplementary Data File 1.csv: The Salmonella phage-bacteria infection matrix

SalmonellaPBIN.Rmd: The R code for the nestedness and modularity analyses

salmonellaPBIN.csv: Input data for the analysis of nestedness and modularity

Supplementary Data File 2: The pairwise comparisons amongst the 13 Salmonella phages
